# Loss of PGRMC1 Delays the Progression of Hepatocellular Carcinoma via Suppression of Pro-Inflammatory Immune Responses

**DOI:** 10.3390/cancers13102438

**Published:** 2021-05-18

**Authors:** Sang R. Lee, Jong Geol Lee, Jun H. Heo, Seong Lae Jo, Jihoon Ryu, Globinna Kim, Jung-Min Yon, Myeong Sup Lee, Geun-Shik Lee, Beum-Soo An, Hyun-Jin Shin, Dong-Cheol Woo, In-Jeoung Baek, Eui-Ju Hong

**Affiliations:** 1 College of Veterinary Medicine, Chungnam National University, Daejeon 34134, Korea; srlee5@naver.com (S.R.L.); heojh94@o.cnu.ac.kr (J.H.H.); jsr7093@o.cnu.ac.kr (S.L.J.); jihoon0511@cnu.ac.kr (J.R.); shin0089@cnu.ac.kr (H.-J.S.); 2 Department of Convergence Medicine, Asan Medical Center, University of Ulsan College of Medicine, Seoul 05505, Korea; superchecker@hanmail.net (J.G.L.); robichi1212@gmail.com (G.K.); yonjungmin@gmail.com (J.-M.Y.); dcwoo@amc.seoul.kr (D.-C.W.); 3 Department of Biomedical Sciences, Asan Medical Center, University of Ulsan College of Medicine, Seoul 05505, Korea; myeong@amc.seoul.kr; 4 College of Veterinary Medicine, Kangwon National University, Chuncheon, Gangwon 24341, Korea; leegeun@kangwon.ac.kr; 5 Department of Biomaterials Science, College of Natural Resources & Life Science, Pusan National University, Miryang, Gyeongsangnam 50463, Korea; anbs@pusan.ac.kr

**Keywords:** *Pgrmc1*, liver cancer, HCC, EGFR, inflammation

## Abstract

**Simple Summary:**

Progesterone receptor membrane component 1 (*PGRMC1*) and epidermal growth factor receptor (*EGFR*) are highly expressed in various cancers. Here, we first analyzed two sets of clinical data and found that the levels of *PGRMC1* and *EGFR* in hepatocellular carcinomas (HCCs) were both inversely correlated with the survival of HCC patients. Accordingly, by using a carcinogen-induced mouse model of HCC, we found that *Pgrmc1* knockout suppressed HCC development and extended the lifespan of HCC-bearing mice. In the acute setting of high-dose carcinogen administration, *Pgrmc1* knockout was associated with increases in hepatic necrosis and decreases in the production of the pro-inflammatory cytokine IL-6. Indeed, silencing of *Pgrmc1* in murine macrophages suppressed IL-6 production and NF-κB activity, and this process was significantly mediated by EGFR. Our study shows that *Pgrmc1* affects the development of HCCs by regulating the EGFR-mediated inflammatory responses. Pgrmc1 may serve as a biomarker and a therapeutic target of HCC.

**Abstract:**

Pgrmc1 is a non-canonical progesterone receptor related to the lethality of various types of cancer. PGRMC1 has been reported to exist in co-precipitated protein complexes with epidermal growth factor receptor (EGFR), which is considered a useful therapeutic target in hepatocellular carcinoma (HCC). Here, we investigated whether *Pgrmc1* is involved in HCC progression. In clinical datasets, *PGRMC1* transcription level was positively correlated with *EGFR* levels; importantly, *PGRMC1* level was inversely correlated with the survival duration of HCC patients. In a diethylnitrosamine (DEN)-induced murine model of HCC, the global ablation of *Pgrmc1* suppressed the development of HCC and prolonged the survival of HCC-bearing mice. We further found that increases in hepatocyte death and suppression of compensatory proliferation in the livers of DEN-injured *Pgrmc1*-null mice were concomitant with decreases in nuclear factor κB (NF-κB)-dependent production of interleukin-6 (IL-6). Indeed, silencing of *Pgrmc1* in murine macrophages led to reductions in NF-κB activity and IL-6 production. We found that the anti-proinflammatory effect of *Pgrmc1* loss was mediated by reductions in EGFR level and its effect was not observed after exposure of the EGFR inhibitor erlotinib. This study reveals a novel cooperative role of *Pgrmc1* in supporting the EGFR-mediated development of hepatocellular carcinoma, implying that pharmacological suppression of *Pgrmc1* may be a useful strategy in HCC treatment.

## 1. Introduction

Liver cancer is one of the leading causes of cancer-related mortality worldwide, and hepatocellular carcinoma (HCC) accounts for approximately 80% of the primary malignant tumor of the liver [1]. The development of HCC is related to chronic inflammation of the liver induced by infection with the hepatitis B and/or C virus, as well as risk factors including aflatoxin exposure, heavy alcohol intake, and non-alcoholic fatty liver disease [2,3]. Since most HCC cases are discovered at an advanced stage, the prognosis of HCC remains poor [4,5]. For HCC patients with progression or in whom local treatment is not feasible, various types of tyrosine kinase inhibitors (TKIs) have been tested as systemic therapy for HCC [6]. As of 2021, sorafenib and lenvatinib are the only two FDA-approved TKIs for use as first-line agents for advanced HCC [7,8,9]; however, those therapies only prolong the survival period by a few months [7,8,9].

Epidermal growth factor receptor (EGFR) has emerged as an important therapeutic target because its overexpression is frequently observed in clinical samples of HCC [10,11]. Moreover, the inhibitors of EGFR such as gefitinib have been proven effective in both in vitro and in vivo models of HCC [12,13]. Although no EGFR-targeted HCC treatments are presently available for clinical use [14,15,16,17], EGFR TKIs showed their synergistic benefit with sorafenib therapy through in vitro and in vivo studies [18,19], and the possible role of EGFR TKIs for the improvement of the efficacy of sorafenib in HCC is being assessed in clinical trials [19].

Progesterone receptor membrane component 1 (Pgrmc1) (Nomenclature: mouse gene: *Pgrmc1*, human gene: *PGRMC1*, zebrafish gene *pgrmc1*, protein: PGRMC1) belongs to the membrane-associated progesterone receptor (MAPR) gene family and contains the N-terminal transmembrane domain and C-terminal cytochrome b5-like heme-binding domain [20]. Since it mediates progesterone’s actions, abnormal phenotypes have been reported in *Pgrmc1* female mutants: Pgr cre-mediated *Pgrmc1* conditional knockout (cKO) in female reproductive tracts exhibited an impaired uterine environment in mice with its ovarian function left normal [21], while the subfertility in *pgrmc1* global KO zebrafish resulted from an impaired ovarian function [22,23], and their uterine- and ovarian-specific abnormalities were seen also in *Pgrmc1*/2 cKO mice [24] and *pgrmc1/2* global KO zebrafish [22,23], respectively. Moreover, endonuclease mediated global *Pgrmc1* KO mice showed a defective development of mammary gland [25]. These phenotypic discrepancies in *Pgrmc1* KO mutants may be due to the differences in model organisms, gene knockout strategy, and organ-specific roles of PGRMC1; nevertheless, it is obvious that PGRMC1 plays a critical role in female reproduction. Beside its classical function such as steroid hormone synthesis [26,27] and metabolic function as a non-genomic progesterone receptor [28,29,30,31], the relevance between *Pgrmc1* and cancers has been suggested in diverse organs. One example is the role of *Pgrmc1* in breast cancer [32,33], as *Pgrmc1* has been recently identified to play important roles in the survival duration and metastasis of breast cancers in a murine PyMT model [34]. *Pgrmc1* is also known to be highly expressed in various types of cancers and associated with the survival rate of patients with head and neck cancer [35], lung and ovarian cancers [36], and breast cancer [32,33]. Of note, PGRMC1 exists in protein complexes including EGFR and modulates its expression levels in multiple cancer cell lines (breast, lung, colon cancers) and affects their proliferation and chemoresistance [20,37]. Likewise, it was demonstrated that *Pgrmc1* positively regulates tumorigenic features of breast cancer cells through the EGFR signaling pathway [38]. While the roles of *Pgrmc1* and the EGFR-mediated signaling pathway have been extensively studied in a variety of cancers, the role of *Pgrmc1*, its signaling pathway, and interaction with EGFR remain poorly understood in the context of HCC.

In this study, we analyzed two clinical datasets and found that low *PGRMC1* and *EGFR* transcripts were associated with longer overall survival durations in HCC-bearing patients and that most patients expressing low *PGRMC1* had low *EGFR* expression as well. We also performed a *Pgrmc1* KO study using a murine model of HCC using diethylnitrosamine (DEN) administration and show that the loss of *Pgrmc1* suppresses the development of HCC and extends the HCC-related survival period. Through further in vitro studies, we delineate the role of *Pgrmc1* in liver macrophages in regulating the pro-inflammatory responses and subsequent development of HCC and show that this process is mediated by EGFR.

## 2. Results

### 2.1. Low PGRMC1 Transcription Level Is Correlated with Extended Survival and Low EGFR Transcription in HCC

To investigate the clinical relevance between *PGRMC1* and HCC, *PGRMC1* transcription levels were analyzed in two gene expression omnibus (GEO) datasets (GSE76427 and GSE20140) which were chosen by matching with previous findings on EGFR and HCC [12,13].

**Figure 1:**
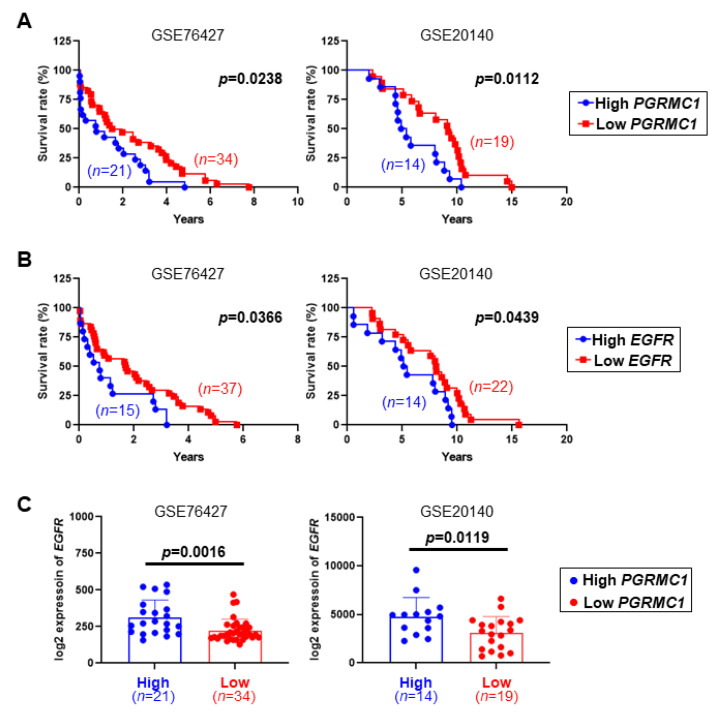
Correlation of *PGRMC1* and *EGFR* with survival duration in patients with hepatocellular carcinoma (HCC). (**A**,**B**) Kaplan–Meier curves for the relationships of the mRNA levels of (**A**) *PGRMC1* and (**B**) *EGFR* with the overall survival duration of patients with hepatocellular carcinoma in two independent cohorts from the GEO database, GSE76427 (left) and GSE20140 (right). (**C**) *EGFR* mRNA expression levels in HCC patients with high or low levels of *PGRMC1* in the GSE76427 (left) and GSE20140 (right) cohorts.

The two datasets showed similar tendencies in which patients with HCCs with a low expression of *PGRMC1* mRNA had a significantly longer survival duration compared with those with HCCs with a high *PGRMC1* expression (Figure 1A); similarly, low *EGFR* expression in the HCCs was associated with prolonged survival (Figure 1B). Moreover, the *EGFR* mRNA expression was significantly lower in the low *PGRMC1* group compared with the high *PGRMC1* group in both datasets (Figure 1C). These data indicate that higher *PGRMC1* expression is significantly associated with worse overall survival in patients with HCC and imply the positive correlation of *PGRMC1* and *EGFR* in HCCs.

### 2.2. Loss of Pgrmc1 Suppresses the Progression and Lethality of HCC in a Murine Model

**Figure 2:**
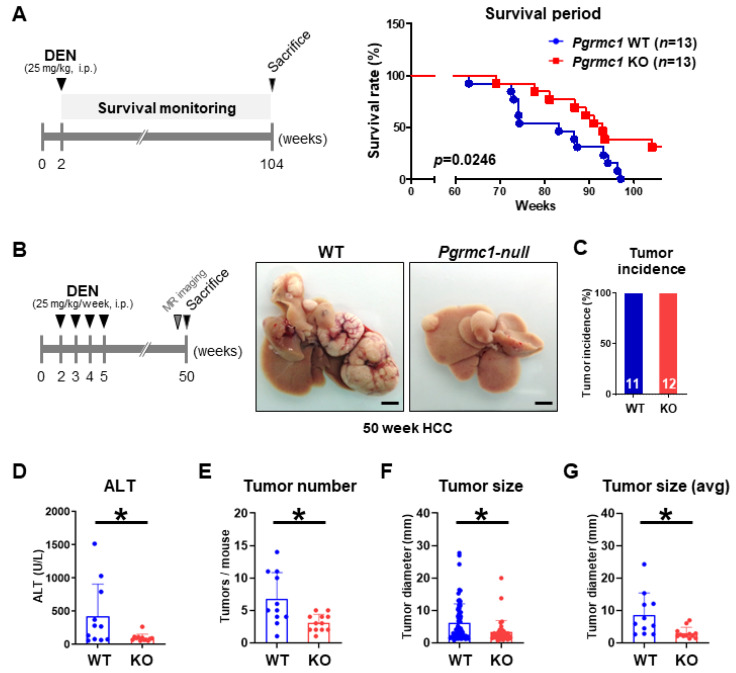
Loss of *Pgrmc1* extends the survival duration and suppresses the development of mice bearing hepatocellular carcinoma (HCC). (**A**) Kaplan–Meier curve of wild-type (WT) and *Pgrmc1* knockout (KO) mice bearing HCC. For the induction of HCC, mice were injected with diethylnitrosamine (DEN) (25 mg/kg, i.p.) at 2-week-old of age, and their survival rate was monitored until 104 weeks. (**B**) Gross images of the livers of WT and *Pgrmc1*-null mice with DEN-induced HCC. Mice (2-weeks-old) were injected with DEN (25 mg/kg/week, i.p.) once a week for 4 weeks and sacrificed at 50 weeks for tumor assessment. Scale bar, 0.5 cm. (**C**) Tumor incidence at 50-weeks-old WT (*n* = 11) and *Pgrmc1* KO (*n* = 12) mice with DEN-induced HCC. (**D**) Plasma ALT level in 50-weeks-old WT and *Pgrmc1* KO mice with DEN-induced HCC. * *p* < 0.05 in Student’s *t*-test. (**E**) Tumor number, (**F**) individual tumor size, and (**G**) average tumor size in the livers of 50-week-old WT and *Pgrmc1* KO mice with DEN-induced HCC. * *p* < 0.05 in Student’s *t*-test.

To experimentally determine the effect of *Pgrmc1* in the survival in a murine model of HCC, we administered mice with diethylnitrosamine (DEN) at 2 weeks of age to induce the development of HCC and monitored their survival for 104 weeks. As a result, we found that *Pgrmc1*-null mice had a significantly longer survival compared with WT mice (Figure 2A). We also examined the effect of *Pgrmc1* in the development and growth of HCCs at 50 weeks of age by administering DEN four times a week in mice since 2 weeks of age (Figure 2B). While all mice in the two groups developed HCCs (Figure 2C), the plasma level of alanine aminotransferase (ALT), a liver injury marker, was significantly lower in *Pgrmc1*-null mice (Figure 2D). Importantly, *Pgrmc1*-null mice developed significantly fewer tumors compared with WT mice (Figure 2E); moreover, the individual size (Figure 2F) and average size (Figure 2G) of the tumors were significantly smaller in *Pgrmc1*-null mice, which was in accordance with their MRI findings (Figure S1). We also assessed the expression level of glypican-3 (GPC3), a biomarker for diagnosis and prognosis of HCC [39], to address whether *Pgrmc1* modulates HCC malignancy in vivo, and found that the GPC3 expression level in the tumor regions was comparable between WT and *Pgrmc1*-null livers (Figure S2). These results show that *Pgrmc1* deficiency suppresses DEN-induced HCC development and extends the survival of mice regardless of its malignancy.

### 2.3. Loss of Pgrmc1 Suppresses Compensatory Proliferation in DEN-Induced Liver Injury

**Figure 3:**
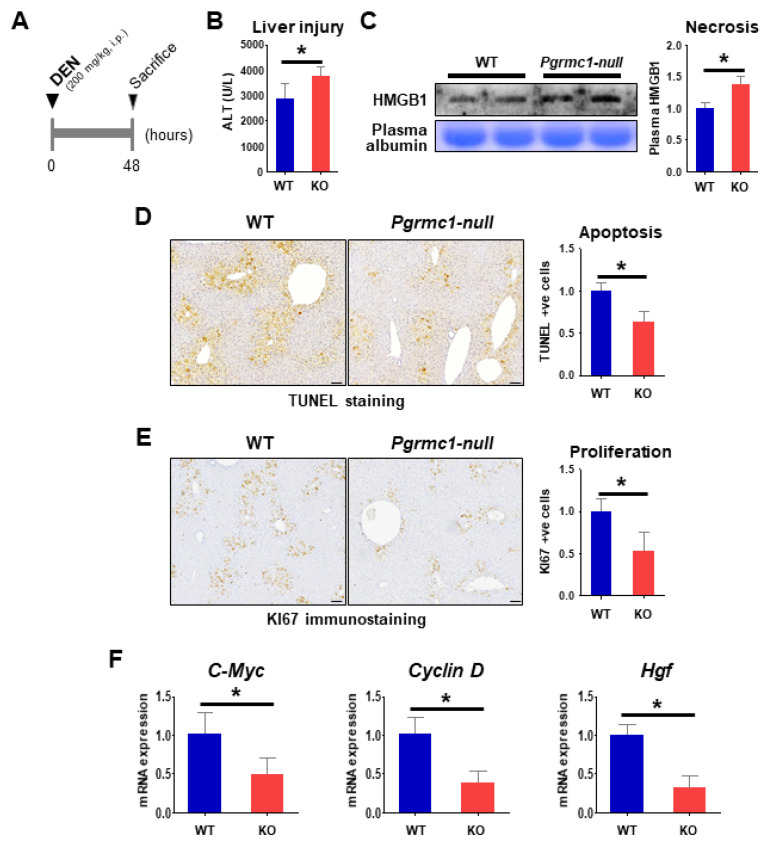
Loss of *Pgrmc1* aggravates liver injury while suppressing compensatory proliferation. (**A**) WT and *Pgrmc1*-null mice were injected with high-dose DEN (200 mg/kg, i.p.), and their plasma and livers were collected after 48 h. (**B**) Plasma level of alanine aminotransferase (ALT). (**C**) Expression of high mobility group box 1 (HMGB1) in the plasma. The expression level was normalized to that of plasma albumin and expressed relative to the WT group. (**D**) TUNEL staining in the livers of WT and *Pgrmc1*-null mice. TUNEL-positive cells were counted and expressed relative to the WT group. Scale bar, 100 μm. (**E**) Ki67 immunostaining in the livers of WT and *Pgrmc1*-null mice. Ki67-positive cells were counted and expressed relative to the WT group. Scale bar, 100 μm. (**F**) mRNA expression levels of *C-Myc*, *Cyclin D*, and *Hgf* in the livers of WT and *Pgrmc1*-null mice. The expression level was normalized to that of *Rplp0* and expressed relative to the WT group. * *p* < 0.05 in Student’s *t*-test. All full blot images were provided in Supplementary File (Figure S3).

Compensatory proliferation, which immediately follows the initial hepatic injury, has a critical role in tumor formation in DEN-induced hepatocarcinogenesis [40,41]. Hence, we investigated the extent of liver injury and proliferation at 48 h after the administration of a high dose of DEN (200 mg/kg i.p.) in 8-weeks-old mice (Figure 3A).

Contrary to the results from the 50-weeks-old HCC-bearing condition, the plasma levels of ALT (Figure 3B) and HMGB1 (Figure 3C) were higher in the short-term DEN-injured *Pgrmc1*-null mice compared with WT, indicating the exacerbation of hepatic injury in *Pgrmc1*-null mice. Increased necrosis of *Pgrmc1*-null mice led to the induction of lymphoid chemotactic factors (Figure S4), while *Pgrmc1*-null HCC did not resemble the trend of acute DEN-injury (Figure S5A).

In contrast, the level of apoptosis, as indicated by TUNEL-positive cells, was rather suppressed in the *Pgrmc1*-null liver as compared to WT (Figure 3D). In addition, *Pgrmc1*-null livers displayed a marked reduction in Ki67-positive signals (Figure 3E) and mRNA expression levels of *C-Myc, Cyclin D*, and *Hgf* (Figure 3F), which are proliferation mediators of HCC cells [42,43,44]. These results showed that *Pgrmc1* ablation suppresses the compensatory proliferation in DEN-induced hepatic injury.

### 2.4. Loss of Pgrmc1 Suppresses EGFR Activation

**Figure 4:**
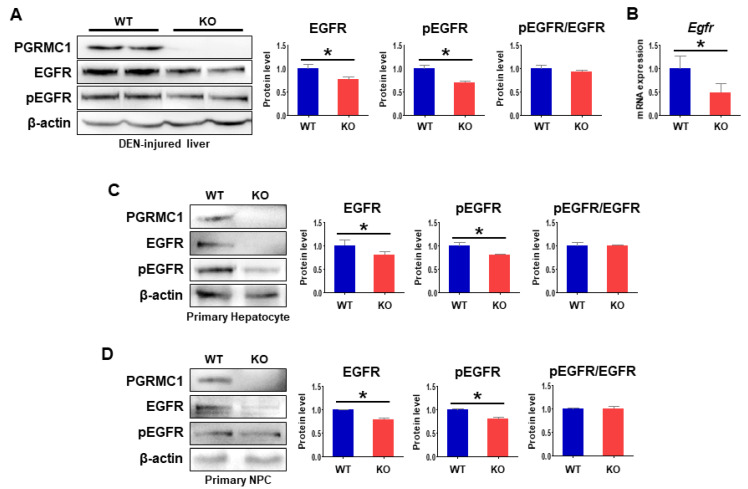
Loss of *Pgrmc1* suppresses EGFR activation. (**A**) PGRMC1, EGFR, and pEGFR expression levels in DEN-injected livers of WT and *Pgrmc1-null* mice. The expression levels were normalized to that of β-actin and expressed relative to the WT group. (**B**) mRNA expression of *Egfr* in DEN-injected livers of WT and *Pgrmc1-null* mice. The expression levels were normalized to that of *Rplp0* and expressed relative to the WT group. * *p* < 0.05 in Student’s *t*-test. (**C**,**D**) PGRMC1, EGFR, and pEGFR expression levels in primary hepatocytes (**C**) and primary non-parenchymal cells (**D**) from WT and *Pgrmc1*-null mice. The expression levels were normalized to that of β-actin and expressed relative to the WT group. * *p* < 0.05 in Student’s *t*-test. All full blot images were provided in Supplementary File (Figure S3).

The GEO analysis in the present study revealed a significant association between *PGRMC1* and *EGFR* in HCC (Figure 1). To determine whether the decreases in HCC development in the *Pgrmc1*-null liver occurred in an EGFR-dependent manner, we analyzed the expression levels of EGFR and its phosphorylation state. We found that the protein levels of EGFR and pEGFR in DEN-injured livers were significantly lower in *Pgrmc1*-null mice than those of WT mice, but that pEGFR/EGFR ratio in *Pgrmc1*-null mice was similar to those of WT mice (Figure 4A). According to these results, *Egfr* mRNA expression was significantly suppressed in DEN-injured livers of *Pgrmc1*-null mice (Figure 4B), suggesting possible regulation of *Egfr* transcription by PGRMC1.

To further determine whether this phenomenon originated from hepatocytes or hepatic immune cells, we separated liver tissues into primary hepatocytes and primary non-parenchymal cells (NPCs). We found that the protein levels of both EGFR and pEGFR were significantly lower in *Pgrmc1*-null primary hepatocytes compared with those of WT mice (Figure 4C), and the same trend was noted in *Pgrmc1*-null primary NPCs (Figure 4D). We also confirmed that pEGFR/EGFR ratio in *Pgrmc1*-null mice was similar to those of WT mice (Figure 4C,D).

### 2.5. Loss of Pgrmc1 Suppresses Pro-Inflammatory Response via EGFR Expression

**Figure 5:**
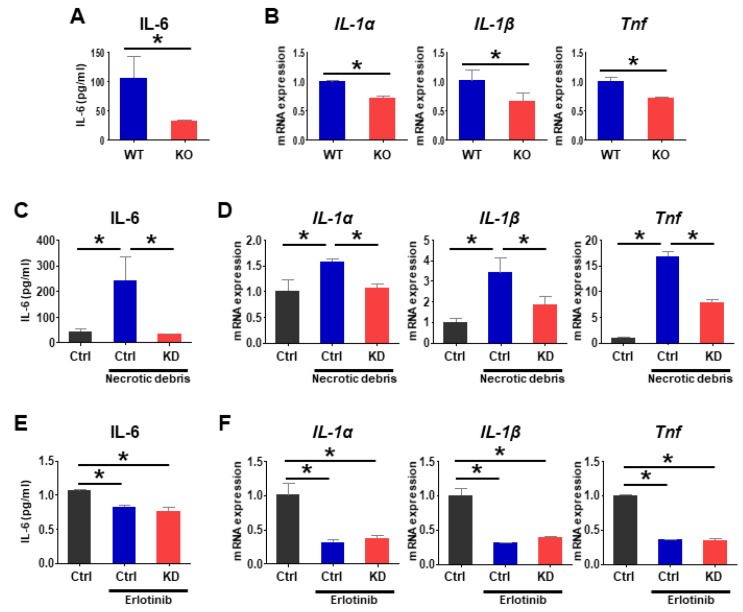
Loss of *Pgrmc1* suppresses IL-6 production in macrophages. (**A**,**B**) Plasma level of IL-6 (**A**) and hepatic mRNA levels of *IL-1α*, *IL-1β*, and *Tnf* (**B**) in DEN-injected WT and *Pgrmc1*-null mice. The mRNA expression levels were normalized to that of *Rplp0* and expressed relative to the WT group. * *p* < 0.05 in Student’s *t*-test. (**C**,**D**) Culture supernatant level of IL-6 (**C**) and mRNA expression levels of *IL-1α*, *IL-1β*, and *Tnf* (**D**) in Raw 264.7 cells transfected with *Control* (Ctrl) or *Pgrmc1*-siRNA (KD) and treated with or without necrotic debris for 3 h. The mRNA expression levels were normalized to that of *Rplp0* and expressed relative to the *Ctrl* group. * *p* < 0.05 in one-way ANOVA followed by Tukey’s post hoc test. (**E**,**F**) *Control* (Ctrl) or *Pgrmc1*-siRNA (KD)-transfected cells were treated with erlotinib (50 μM) and necrotic debris for 8 h. Level of IL-6 (**E**) and mRNA expression levels of *IL-1α*, *IL-1β*, and *Tnf* (**F**) in the supernatant of erlotinib-treated Raw 264.7 cells. The mRNA expression levels were normalized to that of *Rplp0* and expressed relative to the *Ctrl* group. * *p* < 0.05 in one-way ANOVA followed by Tukey’s post hoc test.

Following hepatic injury, dying cells release cytokines that activate liver-resident macrophage (Kupffer cells) [45,46], which also produce cytokines and growth factors that promote the development of HCCs [40,47,48]. Specifically, IL-6, IL-1α, and TNF are important mediators for HCC development [40,46,49]. To investigate how *Pgrmc1* ablation suppresses compensatory proliferation, we evaluated the pro-inflammatory responses in DEN-injured livers and macrophage cells (Raw 264.7). First, in DEN-injured *Pgrmc1*-null mice, pro-inflammatory responses were markedly suppressed compared with WT, as evidenced by the lower levels of plasma IL-6 (Figure 5A) and hepatic mRNA expression levels of *IL-1α*, *IL-1β*, and *Tnf* (Figure 5B). Likewise, suppression in mRNA expression of inflammatory cytokines was also observed in *Pgrmc1*-null HCC (Figure S5B).

Next, we tested the hypothesis that *Pgrmc1*-null macrophages play a pivotal role in the suppression of inflammatory cytokine production and compensatory proliferation after hepatic injury. Considering that necrotic debris released by DEN-injured hepatocytes triggers cytokine production and compensatory proliferation [40,47], we introduced mouse macrophage cells to necrotic debris prepared by freezing and thawing human HCC cells (Hep3B cells). When macrophages treated with *Control* siRNA were challenged with necrotic debris, the level of IL-6 produced in the cell culture medium was significantly increased (Figure 5C), which was reversed in cells treated with *Pgrmc1* siRNA (knockdown; KD) (Figure 5C). Likewise, the debris-treated *Control* siRNA group showed increased mRNA expression levels of *IL-1α*, *IL-1β*, and *Tnf* (Figure 5D), which were reversed in the debris-treated *Pgrmc1* siRNA group (Figure 5D). We also observed that treating Hep3B cells (hepatocellular carcinoma cell line) with supernatant of *Pgrmc1* KD Raw264.7 cells led to down-regulation of cancer cell proliferation (Figure S6A). Likewise, the mRNA level of *HGF* was also suppressed in cells incubated with the supernatant of *Pgrmc1* KD Raw264.7 cells (Figure S6B).

To investigate whether *Pgrmc1* regulates pro-inflammatory response via EGFR, we performed co-immunostaining of PGRMC1 and EGFR in HCC samples, and found co-localization of PGRMC1 and EGFR in non-parenchymal cells, rather than HCC cells (Figure S7). Furthermore, we also observed partial co-localization of PGRMC1 and EGFR in Raw 264.7 cell (Figure S8). To evaluate influence of EGFR on PGRMC1, we treated Raw 264.7 cells with erlotinib, a potent EGFR inhibitor, and observed significant suppression of pro-inflammatory responses (Figure 5E,F). After erlotinib treatment, the level of IL-6 in the cell culture medium was not suppressed by *Pgrmc1* knockdown (Figure 5E). Likewise, the mRNA expression levels of pro-inflammatory cytokines were not suppressed in the erlotinib-treated *Pgrmc1* siRNA group (Figure 5F). Taken together, cytokine levels elevated by necrotic cell debris were dependent upon an erlotinib-sensitive and PGRMC1-dependent tyrosine kinase activity, probably EGFR, and were inhibited by erlotinib.

**Figure 6:**
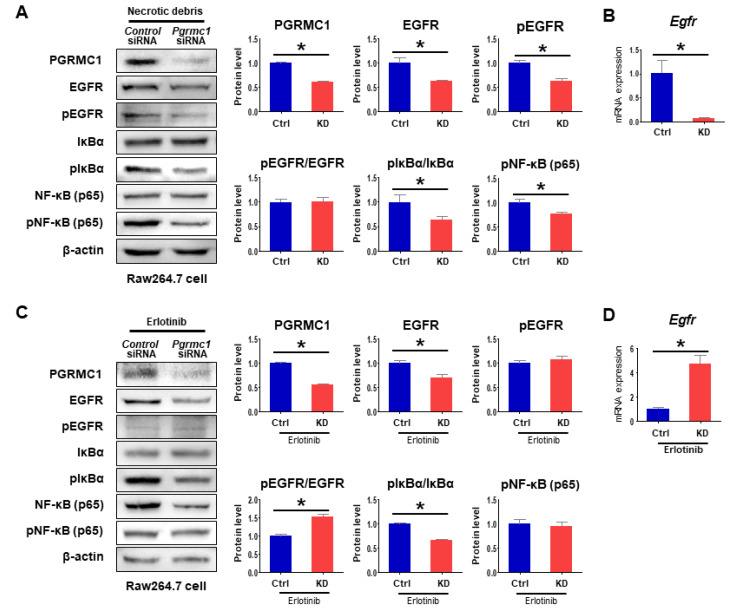
EGFR inhibition by erlotinib nullifies the anti-proinflammatory role of *Pgrmc1* depletion. (**A**) Western blot analysis of PGRMC1, EGFR, pEGFR, IκBα, pIκBα, NF-κB (p65), and pNF-κB (p65) and (**B**) mRNA expression levels of *Egfr* in Raw 264.7 cells transfected with *Control* (Ctrl) or *Pgrmc1*-siRNA (KD) and treated necrotic debris for 3 h. The protein expression levels were normalized to that of β-actin and expressed relative to the *Ctrl* group. The mRNA expression levels were normalized to that of *Rplp0* and expressed relative to the *Ctrl* group. * *p* < 0.05 in Student’s *t*-test. (**C**,**D**) *Control* (Ctrl) or *Pgrmc1*-siRNA (KD)-transfected cells were treated with erlotinib (50 μM) and necrotic debris for 8 h. (**C**) Western blot analysis of PGRMC1, EGFR, pEGFR, IκBα, pIκBα, NF-κB (p65), and pNF-κB (p65) and (**D**) mRNA expression levels of *Egfr* in erlotinib-treated Raw 264.7 cells. The protein expression levels were normalized to that of β-actin and expressed relative to the *Ctrl* group. The mRNA expression levels were normalized to that of *Rplp0* and expressed relative to the *Ctrl* group. * *p* < 0.05 in Student’s *t*-test. All full blot images were provided in Supplementary File (Figure S3).

Considering the reduced percentage of cytokine expression by erlotinib, *Pgrmc1*-KD cells should have a low sensitivity for erlotinib. In Raw 264.7 cells, the protein levels of both EGFR and pEGFR were suppressed in the *Pgrmc1* siRNA group compared with the *Control* siRNA group, while pEGFR/EGFR ratio in *Pgrmc1*-KD cells was similar to those of control cells (Figure 6A). Furthermore, mRNA expression of *Egfr* was suppressed in the *Pgrmc1* siRNA group compared with the *Control* siRNA group (Figure 6B). Notably, while the protein level of EGFR was decreased in the *Pgrmc1* siRNA group, EGFR phosphorylation was similar between the *Control* and *Pgrmc1*-knockdown groups treated with erlotinib (Figure 6C). Therefore, the ratio of pEGFR/EGFR was increased in the *Pgrmc1* siRNA group when compared to control (Figure 6C). The mRNA expression of *Egfr* was also increased by Pgrmc1 knockdown (Figure 6D). These data collectively show that Pgrmc1 positively regulates the expression of EGFR, and that the ablation of Pgrmc1 leads to altered pro-inflammatory responses that may prompt the microenvironment of HCC toward tumor suppression.

After exposure to pro-inflammatory stimuli, activated macrophages produce a panel of inflammatory cytokines and growth factors in an IKK/NF-kB pathway-dependent manner, thereby maintaining liver inflammatory responses and promoting HCC development [45,50]. Accordingly, we investigated the IKK/NF-kB signaling pathway in debris-treated macrophages and found that knockdown of *Pgrmc1* led to decreases in the protein level of pIκBα and the ratio of pIκBα per IκBα (Figure 6A). Likewise, the protein level of pNF-κB (p65) was suppressed in the debris-treated *Pgrmc1* siRNA group (Figure 6A). In erlotinib-treated Raw 264.7 cells, the phosphorylation state of IκBα was significantly lower in the *Pgrmc1*-knockdown group than that of the *Control* siRNA group (Figure 6C), whereas that of NF-κB was not lower in the *Pgrmc1*-knockdown group (Figure 6C). Considering the results of these experiments, it can be postulated that PGRMC1 regulates NF-kB mainly via EGFR. These results collectively suggest that the loss of *Pgrmc1* reduces the pro-inflammatory responses of macrophages in the acute phase of liver injury and thereby contributes to decreased proliferation of hepatocytes in DEN-induced carcinogenesis.

## 3. Discussion

*Pgrmc1* is actively investigated for its possible rols in various cancers, but its relevance in HCC is poorly understood. In the present study, we first analyzed two clinical GEO datasets and found that a low expression of *PGRMC1* mRNA was associated with a longer survival duration in patients with HCC. We further demonstrated that the knockdown of *Pgrmc1* significantly reduced the production of IL-6 in macrophages via the suppression of EGFR in vitro, and that genetic ablation of *Pgrmc1* led to significant decreases in the pro-inflammatory responses after DEN-induced acute hepatic injury in a short-term in vivo study. Importantly, in the long-term in vivo study (>100 weeks), the ablation of *Pgrmc1* led to a notable extension of the survival duration in HCC-bearing mice by suppressing the tumor development. Our findings collectively demonstrate that *Pgrmc1* plays a tumor-promoting function in non-parenchymal cells through the modulation of EGFR expression and may serve as a potential target for treating HCC.

Firstly, we analyzed the TCGA data using an analysis program [51]. Although high *PGRMC1* expression in HCC was correlated with better survival in the TCGA data, the data did not reflect the inflammatory environment of HCC. For example, high *EGFR* expression was not correlated with better survival (*p* = 0.15), while EGFR is related to inflammatory events and targets anti-HCC drugs. Since HCC is a representative example of inflammation-related cancer, its growth was mainly regulated by resident immune cells in the liver than the hepatocytes themselves. When IL-6 was used as an indicator for the inflammatory environment of HCC, *IL-6* expression in the TCGA data did not reflect the inflammatory environment of HCC. Based on these pieces of evidence, we focused on the two clinical data GSE76427 and GSE20140 which reflect inflammation-mediated HCC condition.

DEN, a potent chemical carcinogen, disrupts the cellular DNA of the liver and induces necrosis and apoptosis of hepatocytes [47,52,53]; following the detriment of hepatocytes in the injured liver, the debris of the necrotic cells activates immune cells to produce pro-inflammatory cytokines [54,55] that triggers the compensatory proliferation of hepatocytes, thereby leading to non-resolving inflammation and HCC development [56]. In the present study, although the DEN-induced apoptotic level was decreased in *Pgrmc1*-null livers, the hepatic injury was notably induced by DEN administration as represented by the significantly increased plasma levels of ALT in the *Pgrmc1*-null mice. Moreover, *Pgrmc1*-null livers seemed to be prone to necrosis as evidenced by the increased plasma level of HMGB1, a marker protein released by necrotic cells and not by apoptotic cells [57].

Considering that dead cell debris or necrotic debris is able to activate the pro-inflammatory response by DEN, we speculated that the production of pro-inflammatory cytokines would be enhanced in the *Pgrmc1*-null liver. However, we did not observe a significant degree of compensatory proliferation or proliferative capacity of hepatocytes in *Pgrmc1*-null livers in terms of Ki67 immunostaining and the transcript levels of HCC proliferation markers (i.e., c-*Myc*, *Cyclin* D, and *Hgf*). Considering that a panel of cytokines, chemokines, and growth factors contributes to the crosstalk between tumor and immune cells in the surrounding microenvironment [58], it could be hypothesized that this contradictory phenomenon may arise from the communication between hepatocytes and resident immune cells, which is mediated through inflammatory cytokines or growth factors. Accordingly, the production of inflammatory cytokines (i.e., plasma level of IL-6, hepatic mRNA levels of *IL-1α*, *IL-1β*, and *TNF*) was decreased in *Pgrmc1-null* mice. Therefore, in the context of inflammatory signaling between hepatocytes and immune cells, it can be intermediately concluded that the decreases in compensatory proliferation may result from the reduced amount of cytokine production in the *Pgrmc1*-null liver.

After persistent infection or liver injury, macrophages are one of the major cell types involved in the crosstalk with HCC, especially in the context of a tumor promoter [59]. Specifically, the activation of liver-resident macrophages (Kupffer cells) leads to the recruitment of immune cells including monocytes, which subsequently result in chronic inflammation that shifts the surrounding environment toward a tumor-favoring one [60,61,62,63]. In this study, the necrotic debris of HCC cells (Hep3B cells) induced significant immune responses in macrophages (Raw 264.7 cells) by increasing the amount of pro-inflammatory cytokines. Most importantly, *Pgrmc1* knockdown led to decreases in the plasma level and expression of pro-inflammatory cytokines including IL-6, which is consistent with the decreased level of plasma IL-6 in *Pgrmc1*-null mice. Since macrophage-derived IL-6 plays a pivotal role in the progression of HCCs by regulating proliferation, invasion, and metastasis [40,64], our data suggest that the decreased amount of IL-6 production in *Pgrmc1*-null macrophages would have led to delayed HCC progression. Additionally, *Pgrmc1* knockdown in Raw 264.7 cells also suppressed the phosphorylation of IκBα and NF-κB because various phosphorylation signals were shown to be regulated by PGRMC1 [29]. NF-κB signaling is a major contributor that links inflammation to HCC [65]; moreover, considering its potential relevance with Pgrmc1 in lung adenocarcinoma [66] and breast cancer [38], further targeted studies are needed in order to determine how Pgrmc1 modulates the NF-κB complex in other pathological settings.

In the present study, the genetic ablation or knockdown of *Pgrmc1* suppressed the expression of Egfr at both the protein and transcript levels. In previous reports, it was suggested that PGRMC1 interacts with EGFR via heme-mediated dimerization that requires heme chelation by Y113 [20,37] or alternatively possibly related to PGRMC1 phosphorylation on Y113 [67]. In our results, when the most PGRMC1 was intensely localized in the HCC cells than in the non-parenchymal cells, its presence was not detected in *Pgrmc1*-null livers. Interestingly, EGFR protein was not detected in the HCC cells but in the non-parenchymal cells. EGFR abundance in non-parenchymal cells was decreased in that of *Pgrmc1*-null liver, which suggests a possible relationship between PGRMC1 and EGFR in immune-regulation. To further define the interaction between PGRMC1 and EGFR, we monitored the localization in Raw 264.7 cells expressing PGRMC1 protein and observed the partial co-localization of PGRMC1 and EGFR. These results suggest that limited amount of PGRMC1 in hepatic macrophage could control the EGFR expression involving tumor growth. Further research will be necessary to determine whether PGRMC1:EGFR-containing protein complexes may contribute to these processes.

EGFR, a receptor tyrosine kinase belonging to the ErbB family, is abundantly expressed in the liver and carries a critical function in liver regeneration [68]. EGFR is overexpressed in most clinical HCC cases [11], and its signaling is activated in HCC cells and promotes their growth [69]. As such, the inhibition of EGFR via TKIs such as erlotinib suppresses hepatic fibrosis, cirrhosis, and the development of HCCs [70]. These lines of evidence imply the oncogenic capacity of EGFR in HCC. Interestingly, the differential role of EGFR in different liver cell types has recently been demonstrated in HCC development: Lanaya et al. found that the genetic ablation of *Egfr* in all liver cells or Kupffer cells resulted in impaired hepatocarcinogenesis via decreases in IL-6 production, while hepatocyte-specific knockout of *Egfr* unexpectedly showed the opposite results [71]; these results imply that (1) the role of EGFR in HCC development is different depending on the liver cell types and (2) overall phenotypes resulting from the deletion of *Egfr* both in hepatocytes and macrophages resembles that of macrophage-specific *Egfr* ablation. In the present study, we observed that the knockdown of *Pgrmc1* led to decreased inflammatory responses in macrophages accompanied by downregulation of EGFR, indicating the tumor-promoting role of Pgrmc1 in macrophages. Conversely, a previous in vitro study showed that the knockdown of *PGRMC1* in HCC cells promoted their proliferation while its overexpression caused the opposite effects [72], implying the tumor-suppressing role of Pgrmc1 in hepatocytes. Despite the conflicting roles of Pgrmc1 in hepatocytes and macrophages, *Pgrmc1*-null mice with *Pgrmc1* deficiency in both hepatocytes and macrophages showed suppressed HCC formation, which is in accordance with the phenotypes seen in the aforementioned study by Lanaya et al. on EGFR [71]. PGRMC1 may influence HCC development by modulating EGFR expression apart from EGFR phosphorylation inhibition by erlotinib. Our analysis of two sets of clinical data also showed a similar tendency between *PGRMC1* and *EGFR*. Patients with low transcript levels of *PGRMC1* and *EGFR* in their HCCs had longer survival duration. Furthermore, patients with low *PGRMC1* mRNA expression had low levels of *EGFR* mRNA expression as well. Indeed, we were able to experimentally determine the significant correlation between PGRMC1 and EGFR in an in vitro study using the EGFR inhibitor erlotinib though additional effects of erlotinib on other tyrosine kinase activities cannot be excluded by our data; in cells treated with erlotinib, the anti-proinflammatory effect by *Pgrmc1* knockdown was not observed, suggesting that EGFR inactivation by the loss of *Pgrmc1* is essential for suppression of pro-inflammatory response in this context.

## 4. Materials and Methods

### 4.1. GEO Dataset Analysis

The transcription levels of *PGRMC1* and *EGFR* in clinical samples of HCCs were analyzed using the public GEO dataset (GSE76427, GSE20140). The patients in each dataset were divided into quartiles according to the transcription levels of *PGRMC1* and *EGFR*; of them, patients in the Q1 and Q3 in each dataset were selected for analysis. For *PGRMC1* analysis, the numbers of patients used in the analyses were as follows: 34 out of 34 (low *PGRMC1*) and 21 out of 21 (high *PGRMC1*) in the GSE76427 dataset, and 19 out of 21 (low *PGRMC1*) and 14 out of 16 (high *PGRMC1*) in the GSE20140 dataset. For *EGFR* analysis, the numbers of patients used in the analyses were as follows: 37 out of 38 (low *EGFR*) and 15 out of 16 (high *EGFR*) in the GSE76427 dataset, and 22 out of 24 (low *EGFR*) and 14 out of 15 (high *EGFR*) in the GSE20140 dataset. For the comparison between *PGRMC1* and *EGFR* expression levels, patients were divided according to the *PGRMC1* expression levels.

### 4.2. Animals

All animal experiments were performed in accordance with the Korean Ministry of Food and Drug Safety (MFDS) guidelines, and the protocols were reviewed and approved by the Institutional Animal Care and Use Committee of the Asan Institute for Life Sciences (permit number: 2017-13-139). Only male mice were included in the experiments. WT and *Pgrmc1-null* mice were used according to a previously reported protocol [73]. To induce HCC, 2-week-old mice were administered with DEN (25 mg/kg, intraperitoneal; IP). To induce acute liver injury, 8-week-old mice were administered with a high dose of DEN (200 mg/kg, IP) and sacrificed after 48 h using CO_2_ asphyxiation.

### 4.3. In Vivo Magnetic Resonance Imaging

To monitor the incidence and size of HCC, MR images were acquired using a 9.4 Tesla 160 mm system (Agilent Inc., Palo Alto, CA, USA). Animals were anesthetized through a mask via spontaneous inhalation of 2.0–2.5% isoflurane in a 1:2 mixture of O_2_:N_2_O. Respiration was monitored and the mice were maintained in normothermic conditions at 37.5 ± 0.5 °C using a heating bath circulator (CW-05G Heated Circulating Water Bath, St. Louis, MO, USA). The MRI protocol included T1-weighted (T1w) − TR/TE = 1100/8.93 ms; average = 3; field of view (FOV) = 25 × 35 mm (axial); matrix = 128 × 128; and slice thickness = 1.5 mm; and T2-weighted (T2w) − TR/TE = 4000/10.0 ms; average = 3, FOV = 25 × 35 mm (axial) or 35 × 35 mm (coronal), matrix = 128 × 128 or 256 × 256, and slice thickness = 1.5 mm. The chemical shift selective saturation (CHESS) pulse sequence was used at −1338 Hz to suppress the fat signal. Respiratory/heart gating was used for MRI scanning, and the total MR scan time was within 20 min. Abnormal structures/high signals on MR images were regarded as tumors.

### 4.4. RNA Isolation, Reverse Transcription, and qRT-PCR

RNA was extracted from livers, HCC, and Raw 264.7 cells. After homogenization with the Trizol reagent, homogenates were mixed with chloroform. The mixture was centrifuged, and the supernatant was incubated with isopropanol. RNA pellets were dissolved in DEPC-treated water. cDNA was synthesized with 1 µg of total RNA by using a reverse transcriptase kit (SG-cDNAS100, Smartgene, Daejeon, Korea) according to the manufacturer’s protocol. Quantitative PCR (real-time PCR) was carried out using specific primers (Table 1), Excel Taq Q-PCR Master Mix (SG-SYBR-500, Smartgene, Daejeon, Korea), and Stratagene Mx3000P (Agilent Technologies, CA, USA) equipped with a 96-well optical reaction plate. All experiments were repeated in triplicates, and the mRNA values were calculated based on the cycle threshold and monitored for a melting curve.

### 4.5. Western Blot

Protein was extracted from livers, Raw 264.7 cells, primary hepatocytes, and primary NPCs by homogenization with T-PER buffer. After electrophoresis, gels were blotted to a PVDF membrane, and the membrane was blocked and incubated with primary antibodies. After overnight incubation, the membranes were washed and incubated with secondary antibodies (Goat anti-Rabbit IgG HRP; Catalog # 31460, Goat anti-Mouse IgG HRP; Catalog # 31430, Thermo Fisher Scientific, MA, USA). The bands were observed with ECL solution (XLS025-0000, Cyanagen, BO, Italy) after washing three times.

The following primary antibodies were used: β-actin (sc-130656, Santa Cruz Biotechnology), phosphor-IκBα (pIκBα), NF-κB p65, phosphor-NF-κB p65 (pNF-κB p65) (9936, CST, MA, USA), EGFR (A2909, ABclonal, MA, USA), phosphor-EGFR (9789, CST, MA, USA), HMGB1 (CSB-PA01604A0Rb, Cusabio, TX, USA), PGRMC1 (rabbit monoclonal, 13856, CST, MA, USA), and IκBα (mouse monoclonal, 9936, CST, MA, USA).

### 4.6. Cell Culture and Gene Knockdown

All cell culture reagents were purchased from Welgene (Gyungsan, Korea). Hep3B human liver cancer cells and Raw 264.7 mouse macrophage cells were maintained at 37 °C in a 5% CO_2_ atmosphere in DMEM (LM001-05, Welgene, Gyungsan, Korea) supplemented with 5% (vol/vol) fetal bovine serum, penicillin (100 U/mol), and streptomycin (100 μg/mL). For knockdown of *PGRMC1*, siRNA transfection was performed using the Lipofectamine 2000 reagent (11668-027, Thermo Fisher Scientific, MA, USA) according to the manufacturer’s protocol. Negative control siRNA and *PGRMC1* siRNA #1 and #2 were purchased from Bioneer (Daejeon, Korea). The sense sequences of *PGRMC1* siRNA #1 and #2 were 5′-CAGUACAGUCGCUAGUCAA-3′ and 5′-CAGUUCACUUUCAAGUAUCA-U-3′. Erlotinib hydrochloride (SML2156, Sigma-aldrich, MO, USA) was used for EGFR inhibition. To induce inflammatory response, necrotic cell debris was collected from frozen-melted Hep3B cells and treated to Raw 264.7 cells.

### 4.7. Primary Cell Culture

Mouse livers were perfused with Ca^2+^ and Mg^2+^ free-Hanks’ Balanced Salt Solution (HBSS) containing EDTA (1 mM) via portal vein catheterization, removed from the mice, gently teased with forceps, and digested with a collagenase solution containing liberase (0540119001, Sigma-aldrich, MO, USA). The liver-containing solution was filtered through a sterile 40 μm nylon cell strainer (93040, SPL Life Sciences, Pocheon, Korea) to remove undigested tissues and connective tissues. The cells were centrifuged briefly at 1000 rpm (brake off), and the pellet was suspended in a cell culture medium. The suspended cells were again centrifuged briefly at 1000 rpm, suspended in DMEM supplemented with 5% FBS, and then seeded onto culture plates. Adherent hepatocytes were used for experiments after overnight incubation. For the collection of NPCs, supernatant collected from the first centrifugation was seeded onto culture plates. Non-adherent cells were collected after 2 h and seeded onto another culture plate. The adherent NPCs after overnight incubation were used for experiments.

### 4.8. Alanine Aminotransferase (ALT) Measurement

Plasma was diluted at a 1:5 ratio, and the plasma ALT level was measured with FUJI DRI-CHEM SLIDE (ALT-3250) by DRI-CHEM4000 (Fuji Film, Tokyo, Japan). Values were calculated considering the dilution factor.

### 4.9. Immunostaining

For immunohistochemistry, 4 µm-thick tissue sections were deparaffinized, serially rehydrated with ethanol, subjected to antigen retrieval using a citrate buffer (10 mM citric acid, 0.05% Tween 20, pH 6.0), and then immunostained with the antibody against Ki67 (15580, Abcam, Cambridge, UK) using the VECTASTAIN ABC kit (PK6101, Vector Labs, CA, USA) according to the manufacturer’s instructions. To measure the degree of apoptosis, TUNEL staining was performed using the DeadEnd colorimetric TUNEL system (G7130, Promega, WI, USA) following the manufacturer’s instructions. Ki67- or TUNEL-positive areas per liver were quantified using the ImageJ software (NIH, Bethesda, MD, USA).

For co-staining of PGRMC1 and EGFR in tumor sections, antigen-retrieved paraffin sections were incubated with mouse immunoglobulin blocking reagent (PK2200, Vector Labs, CA, USA) and blocked with 5% goat serum prior to overnight incubation with anti-PGRMC1 (rabbit monoclonal, 13856, CST, MA, USA) and anti-EGFR (mouse monoclonal, sc-373746, Santa Cruz Biotechnology, TX, USA) primary antibodies. The sections were then incubated for 1 h with Alexa Fluor 488-conjugated goat anti-rabbit IgG (A11008, Thermo Fisher Scientific, MA, USA) and subsequently for another 1 h with Alexa Fluor 594-conjugated goat anti-mouse IgG (A11005, Thermo Fisher Scientific, MA, USA). After mounting with DAPI (D1306, Thermo Fisher Scientific, MA, USA), the sections were examined under Zeiss LSM 880 laser scanning confocal microscope system (Carl Zeiss, Oberkochen, Germany).

For co-staining of PGRMC1 and EGFR in Raw 264.7 cell, cells grown in chamber slide (80826, Ibidi, Gräfelfing, Germany) were fixed in cold methanol for 30 min and washed with PBS. Cells were blocked with 3% BSA prior to overnight incubation with anti-PGRMC1 (rabbit polyclonal, A5619, ABclonal, MA, USA) and anti-EGFR (mouse monoclonal, sc-373746, Santa Cruz Biotechnology, TX, USA) primary antibodies. Cells were then incubated for 2 h with anti-rabbit (A21207, Thermo Fisher Scientific, MA, USA) and anti-mouse (A21202, Thermo Fisher Scientific, MA, USA) antibodies. Cells were washed with PBS, treated with DAPI, and washed again for subsequent observation. Region of interest was observed in dark area with microscope.

### 4.10. Enzyme-Linked Immunosorbent Assay (ELISA)

Mouse plasma and cell culture medium were collected for the measurement of IL-6 using ELISA. Samples were processed with a mouse IL-6 ELISA kit (EKC40085, Biomatik) according to the manufacturer’s protocol.

### 4.11. Statistical Analysis

Data are reported as mean ± standard deviation. Differences between the means were assessed using Student’s *t*-test and one-way ANOVA followed by a Tukey’s multiple comparison test. All statistical analyses were performed using the GraphPad Software (GraphPad Inc., San Diego, CA, USA).

## 5. Conclusions

In the current study, we made substantial progress towards delineating the *Pgrmc1* mediated pro-inflammatory mechanism in HCC. In clinical data analysis, the expression level of *PGRMC1* mRNA was inversely correlated to survival duration in patients with HCC, suggesting that *Pgrmc1* mRNA levels may be used as a prognostic marker for the progression of HCC. Furthermore, our data suggest that specific inhibitors of Pgrmc1 have a potential in conferring synergistic effects with the existing anti-HCC drugs. Lastly, our results from HCC-bearing *Pgrmc1*-null mice may be useful in the therapeutic approach for examining the survival duration and the suppression of HCC development in patients. Further research should address which specific interaction or mechanism underlies on PGRMC1:EGFR-containing protein complexes and contributes to HCC.

## Figures and Tables

**Table 1:** Primers used for real-time PCR.

Gene Name	Upper Primer (5′–3′)	Lower Primer (5′–3′)	Species
*IL-1α*	AGT ATC AGC AAC GTC AAG CAA	TCC AGA TCA TGG GTT ATG GAC TG	Mouse
*IL-1β*	GAA ATG CCA CCT TTT GAC AGT G	CTG GAT GCT CTC ATC AGG ACA	Mouse
*TNF*	CCT GTA GCC CAC GTC GTA G	GGG AGT AGA CAA GGT ACA ACC C	Mouse
*Ccl19*	GGG GTG CTA ATG ATG CGG AA	CCT TAG TGT GGT GAA CAC AAC A	Mouse
*Ccl21*	GTG ATG GAG GGG GTC AGG A	GGG ATG GGA CAG CCT AAA CT	Mouse
*Icam1*	GTG ATG CTC AGG TAT CCA TCC A	CAC AGT TCT CAA AGC ACA GCG	Mouse
*F4/80*	TTG TAC GTG CAA CTC AGG ACT	GAT CCC AGA GTG TTG ATG CAA	Mouse
*Egfr*	GCA TCA TGG GAG AGA ACA ACA	TCA GGA ACC ATT ACT CCA TAG GT	Mouse
*Rplp0*	GCA GCA GAT CCG CAT GTC GCT CCG	GAG CTG GCA CAG TGA CCT CAC ACG G	Mouse
*C-Myc*	GCT CTC CAT CCT ATG TTG CGG	TCC AAG TAA CTC GGT CAT CAT CT	Mouse
*Cyclin D*	GCG TAC CCT GAC ACC AAT CTC	CTC CTC TTC GCA CTT CTG CTC	Mouse
*Hgf*	TTC ATG TCG CCA TCC CCT ATG	CCC CTG TTC CTG ATA CAC CT	Mouse
*C-Myc*	CCT ACC CTC TCA ACG ACA GC	CTC TGA CCT TTT GCC AGG AG	Human
*Cyclin D*	GCT GCG AAG TGG AAA CCA TC	CCT CCT TCT GCA CAC ATT TGA A	Human
*Hgf*	CTG GTT CCC CTT CAA TAG CA	AAC TCC AGG GCT GAC ATT TG	Human
*Rplp0*	TCG ACA ATG GCA GCA TCT AC	GCC TTG ACC TTT TCA GCA AG	Human

## Data Availability

The data presented in this study are available on request from the corresponding author.

## Supplementary Materials

The following are available online at https://www.mdpi.com/article/10.3390/cancers13102438/s1, Figure S1. Magnetic resonance images (T1w [axial], T2w [axial and coronal]) of HCCs in HCC-bearing WT (*n* = 11) and Pgrmc1-null (*n* = 12) mice. Arrows denote the tumors, Figure S2. Western blot analysis of Glypican-3 (GPC3) in non-tumor lesions and tumors of WT and Pgrmc1-null mice. β-actin was used as an internal control. NT, non-tumor; T, tumor region, Figure S3. Full blot images, Figure S4. mRNA expression levels of F4/80 (A), Ccl19, Ccl21, and Icam1 (B), in the livers of DEN-injected WT and Pgrmc1-null mice. The expression levels were normalized to that of Rplp0 and expressed relative to the WT group. * *p* < 0.05 in Student’s *t*-test, Figure S5. mRNA expression levels of F4/80, Ccl19, Ccl21, Icam1 (A), IL-1α, IL-1β, and Tnf (B) in the HCC of DEN-injected WT and Pgrmc1-null mice. The expression levels were normalized to that of Rplp0 and expressed relative to the WT group. * *p* < 0.05 in Student’s *t*-test, Figure S6. Cancer cell proliferation measured after treatment of Raw264.7 cell culture supernatant. (A) Cell number counted by trypan blue staining in 0 and 24 h treatment of Raw264.7 cell culture supernatant to Hep3B cells. FBS was excluded in Hep3B cell culture medium. Cell culture supernatant was collected from Control (Ctrl) or Pgrmc1-siRNA (KD)-transfected Raw 264.7 cells treated with necrotic debris for 3 h. (B) mRNA expression levels of C-Myc, Cyclin D, and HGF in Hep3B cells cultured with Raw 264.7 cell culture supernatant. Cell culture supernatant was collected from Control (Ctrl) or Pgrmc1-siRNA (KD)-transfected Raw 264.7 cells treated with necrotic debris for 3 h. Cell culture supernatant was incubated for 3 h in Hep3B cells. * *p* < 0.05 in Student’s *t*-test, Figure S7. Co-immunostaining of PGRMC1 and EGFR in the HCC of WT mice and Pgrmc1-null mice. PGRMC1 (green) and EGFR (red) were stained with the corresponding antibodies. DAPI (blue) was used for nuclear staining, Figure S8. Co-immunostaining of PGRMC1 and EGFR in Raw 264.7 cell. PGRMC1 (red) and EGFR (green) were stained with the corresponding antibodies. DAPI (blue) was used for nuclear staining.

## References

[1] Sung H., Ferlay J., Siegel R.L., Laversanne M., Soerjomataram I., Jemal A., Bray F. (2021). Global cancer statistics 2020: GLOBOCAN estimates of incidence and mortality worldwide for 36 cancers in 185 countries. CA Cancer J. Clin..

[2] Tu T., Bühler S., Bartenschlager R. (2017). Chronic viral hepatitis and its association with liver cancer. Biol. Chem..

[3] Capece D., Fischietti M., Verzella D., Gaggiano A., Cicciarelli G., Tessitore A., Zazzeroni F., Alesse E. (2013). The inflammatory microenvironment in hepatocellular carcinoma: A pivotal role for tumor-associated macrophages. Biomed. Res. Int..

[4] Forner A., Reig M., Bruix J. (2018). Hepatocellular carcinoma. Lancet.

[5] Kulik L., El-Serag H.B. (2019). Epidemiology and Management of Hepatocellular Carcinoma. Gastroenterology.

[6] Hasskarl J. (2014). Sorafenib: Targeting multiple tyrosine kinases in cancer. Recent Results Cancer Res..

[7] Llovet J.M., Ricci S., Mazzaferro V., Hilgard P., Gane E., Blanc J.F., De Oliveira A.C., Santoro A., Raoul J.L., Forner A. (2008). Sorafenib in advanced hepatocellular carcinoma. N. Engl. J. Med..

[8] Kudo M., Finn R.S., Qin S., Han K.-H., Ikeda K., Piscaglia F., Baron A., Park J.-W., Han G., Jassem J. (2018). Lenvatinib versus sorafenib in first-line treatment of patients with unresectable hepatocellular carcinoma: A randomised phase 3 non-inferiority trial. Lancet.

[9] Cheng A.-L., Kang Y.-K., Chen Z., Tsao C.-J., Qin S., Kim J.S., Luo R., Feng J., Ye S., Yang T.-S. (2009). Efficacy and safety of sorafenib in patients in the Asia-Pacific region with advanced hepatocellular carcinoma: A phase III randomised, double-blind, placebo-controlled trial. Lancet Oncol..

[10] Ito Y., Takeda T., Sakon M., Tsujimoto M., Higashiyama S., Noda K., Miyoshi E., Monden M., Matsuura N. (2001). Expression and clinical significance of erb-B receptor family in hepatocellular carcinoma. Br. J. Cancer.

[11] Buckley A.F., Burgart L.J., Sahai V., Kakar S. (2008). Epidermal growth factor receptor expression and gene copy number in conventional hepatocellular carcinoma. Am. J. Clin. Pathol..

[12] Höpfner M., Sutter A.P., Huether A., Schuppan D., Zeitz M., Scherübl H. (2004). Targeting the epidermal growth factor receptor by gefitinib for treatment of hepatocellular carcinoma. J. Hepatol..

[13] Schiffer E., Housset C., Cacheux W., Wendum D., Rey C., Poupon R., Rosmorduc O. (2005). Gefitinib, an EGFR inhibitor, prevents hepatocellular carcinoma development in the rat liver with cirrhosis. Hepatology.

[14] Whittaker S., Marais R., Zhu A.X. (2010). The role of signaling pathways in the development and treatment of hepatocellular carcinoma. Oncogene.

[15] Philip P.A., Mahoney M.R., Allmer C., Thomas J., Pitot H.C., Kim G., Donehower R.C., Fitch T., Picus J., Erlichman C. (2005). Phase II study of Erlotinib (OSI-774) in patients with advanced hepatocellular cancer. J. Clin. Oncol..

[16] Thomas M.B., Chadha R., Glover K., Wang X., Morris J., Brown T., Rashid A., Dancey J., Abbruzzese J.L. (2007). Phase 2 study of erlotinib in patients with unresectable hepatocellular carcinoma. Cancer.

[17] Zhu A.X., Stuart K., Blaszkowsky L.S., Muzikansky A., Reitberg D.P., Clark J.W., Enzinger P.C., Bhargava P., Meyerhardt J.A., Horgan K. (2007). Phase 2 study of cetuximab in patients with advanced hepatocellular carcinoma. Cancer.

[18] Ezzoukhry Z., Louandre C., Trécherel E., Godin C., Chauffert B., Dupont S., Diouf M., Barbare J.-C., Mazière J.-C., Galmiche A. (2012). EGFR activation is a potential determinant of primary resistance of hepatocellular carcinoma cells to sorafenib. Int. J. Cancer.

[19] Lin C.-H., Elkholy K.H., Wani N.A., Li D., Hu P., Barajas J.M., Yu L., Zhang X., Jacob S.T., Khan W.N. (2020). Ibrutinib Potentiates Antihepatocarcinogenic Efficacy of Sorafenib by Targeting EGFR in Tumor Cells and BTK in Immune Cells in the Stroma. Mol. Cancer Ther..

[20] Kabe Y., Nakane T., Koike I., Yamamoto T., Sugiura Y., Harada E., Sugase K., Shimamura T., Ohmura M., Muraoka K. (2016). Haem-dependent dimerization of PGRMC1/Sigma-2 receptor facilitates cancer proliferation and chemoresistance. Nat. Commun..

[21] McCallum M.L., Pru C.A., Niikura Y., Yee S.-P., Lydon J.P., Peluso J.J., Pru J.K. (2016). Conditional Ablation of Progesterone Receptor Membrane Component 1 Results in Subfertility in the Female and Development of Endometrial Cysts. Endocrinology.

[22] Wu X.-J., Zhu Y. (2020). Downregulation of nuclear progestin receptor (Pgr) and subfertility in double knockouts of progestin receptor membrane component 1 (pgrmc1) and pgrmc2 in zebrafish. Gen. Comp. Endocrinol..

[23] Wu X.-J., Thomas P., Zhu Y. (2018). Pgrmc1 Knockout Impairs Oocyte Maturation in Zebrafish. Front. Endocrinol..

[24] Clark N.C., Pru C.A., Yee S.-P., Lydon J.P., Peluso J.J., Pru J.K. (2017). Conditional Ablation of Progesterone Receptor Membrane Component 2 Causes Female Premature Reproductive Senescence. Endocrinology.

[25] Kim G., Lee J.G., Cheong S.-A., Yon J.-M., Lee M.S., Hong E.-J., Baek I.-J. (2020). Progesterone receptor membrane component 1 is required for mammary gland developmentdagger. Biol. Reprod..

[26] Lee S.R., Yang H., Jo S.L., Lee Y.H., Lee H.W., Park B.-K., Hong E.-J. (2021). Suppressed estrogen supply via extra-ovarian progesterone receptor membrane component 1 in menopause. J. Biomed. Res..

[27] Cahill M.A., Medlock A.E. (2017). Thoughts on interactions between PGRMC1 and diverse attested and potential hydrophobic ligands. J. Steroid Biochem. Mol. Biol..

[28] Thejer B.M., Adhikary P.P., Teakel S.L., Fang J., Weston P.A., Gurusinghe S., Anwer A.G., Gosnell M., Jazayeri J.A., Ludescher M. (2020). PGRMC1 effects on metabolism, genomic mutation and CpG methylation imply crucial roles in animal biology and disease. BMC Mol. Cell Biol..

[29] Thejer B.M., Adhikary P.P., Kaur A., Teakel S.L., Van Oosterum A., Seth I., Pajic M., Hannan K.M., Pavy M., Poh P. (2020). PGRMC1 phosphorylation affects cell shape, motility, glycolysis, mitochondrial form and function, and tumor growth. BMC Mol. Cell Biol..

[30] Lee S.R., Heo J.H., Jo S.L., Kim G., Kim S.J., Yoo H.J., Lee K.-P., Kwun H.-J., Shin H.-J., Baek I.-J. (2021). Progesterone receptor membrane component 1 reduces cardiac steatosis and lipotoxicity via activation of fatty acid oxidation and mitochondrial respiration. Sci. Rep..

[31] Lee S.R., Choi W.-Y., Heo J.H., Huh J., Kim G., Lee K.-P., Kwun H.-J., Shin H.-J., Baek I.-J., Hong E.-J. (2020). Progesterone increases blood glucose via hepatic progesterone receptor membrane component 1 under limited or impaired action of insulin. Sci. Rep..

[32] Cai G., Yang X., Ruan X., Wang J., Fang Y., Wei Y., Zhang Y., Gu M., Mueck A.O. (2020). Association of circulating Progesterone Receptor Membrane Component-1 (PGRMC1) with PGRMC1 expression in breast tumour tissue and with clinical breast tumour characteristics. Maturitas.

[33] Ruan X., Zhang Y., Mueck A.O., Willibald M., Seeger H., Fehm T., Brucker S., Neubauer H. (2017). Increased expression of progesterone receptor membrane component 1 is associated with aggressive phenotype and poor prognosis in ER-positive and negative breast cancer. Menopause.

[34] Lee S.R., Lee Y.H., Jo S.L., Heo J.H., Kim G., Lee G.-S., An B.-S., Baek I.-J., Hong E.-J. (2021). Absence of progesterone receptor membrane component 1 reduces migration and metastasis of breast cancer. Cell Commun. Signal..

[35] Zhao Y., Ruan X. (2019). Identification of PGRMC1 as a Candidate Oncogene for Head and Neck Cancers and Its Involvement in Metabolic Activities. Front. Bioeng. Biotechnol..

[36] Hampton K.K., Stewart R., Napier D., Claudio P.P., Craven R.J. (2015). PGRMC1 Elevation in Multiple Cancers and Essential Role in Stem Cell Survival. Adv. Lung Cancer.

[37] Ahmed I.S., Rohe H.J., Twist K.E., Craven R.J. (2010). Pgrmc1 (progesterone receptor membrane component 1) associates with epidermal growth factor receptor and regulates erlotinib sensitivity. J. Biol. Chem..

[38] Pedroza D.A., Rajamanickam V., Subramani R., Bencomo A., Galvez A., Lakshmanaswamy R. (2020). Progesterone receptor membrane component 1 promotes the growth of breast cancers by altering the phosphoproteome and augmenting EGFR/PI3K/AKT signalling. Br. J. Cancer.

[39] Zhou F., Shang W., Yu X., Tian J. (2018). Glypican-3: A promising biomarker for hepatocellular carcinoma diagnosis and treatment. Med. Res. Rev..

[40] Naugler W.E., Sakurai T., Kim S., Maeda S., Kim K., Elsharkawy A.M., Karin M. (2007). Gender disparity in liver cancer due to sex differences in MyD88-dependent IL-6 production. Science.

[41] Shang N., Bank T., Ding X., Breslin P., Li J., Shi B., Qiu W. (2018). Caspase-3 suppresses diethylnitrosamine-induced hepatocyte death, compensatory proliferation and hepatocarcinogenesis through inhibiting p38 activation. Cell Death Dis..

[42] Goyal L., Muzumdar M.D., Zhu A.X. (2013). Targeting the HGF/c-MET pathway in hepatocellular carcinoma. Clin. Cancer Res..

[43] Chen J., Li X., Cheng Q., Ning D., Ma J., Zhang Z., Chen X., Jiang L. (2018). Effects of cyclin D1 gene silencing on cell proliferation, cell cycle, and apoptosis of hepatocellular carcinoma cells. J. Cell Biochem..

[44] Qu A., Jiang C., Cai Y., Kim J.-H., Tanaka N., Ward J.M., Shah Y.M., Gonzalez F.J. (2014). Role of Myc in hepatocellular proliferation and hepatocarcinogenesis. J. Hepatol..

[45] He G., Karin M. (2011). NF-kappaB and STAT3—Key players in liver inflammation and cancer. Cell Res..

[46] Sakurai T., He G., Matsuzawa A., Yu G.-Y., Maeda S., Hardiman G., Karin M. (2008). Hepatocyte necrosis induced by oxidative stress and IL-1 alpha release mediate carcinogen-induced compensatory proliferation and liver tumorigenesis. Cancer Cell.

[47] Maeda S., Kamata H., Luo J.-L., Leffert H., Karin M. (2005). IKKbeta couples hepatocyte death to cytokine-driven compensatory proliferation that promotes chemical hepatocarcinogenesis. Cell.

[48] Schwabe R.F., Brenner D.A. (2006). Mechanisms of Liver Injury. I. TNF-alpha-induced liver injury: Role of IKK, JNK, and ROS pathways. Am. J. Physiol. Liver Physiol..

[49] Jing Y., Sun K., Liu W., Sheng D., Zhao S., Gao L., Wei L. (2018). Tumor necrosis factor-alpha promotes hepatocellular carcinogenesis through the activation of hepatic progenitor cells. Cancer Lett..

[50] Luedde T., Schwabe R.F. (2011). NF-kappaB in the liver--linking injury, fibrosis and hepatocellular carcinoma. Nat. Rev. Gastroenterol. Hepatol..

[51] Lian Q., Wang S., Zhang G., Wang D., Luo G., Tang J., Chen L., Gu J. (2018). HCCDB: A Database of Hepatocellular Carcinoma Expression Atlas. Genom. Proteom. Bioinform..

[52] Tessitore L. (2000). Apoptosis and cell proliferation are involved in the initiation of liver carcinogenesis by a subnecrogenic dose of diethylnitrosamine in refed rats. J. Nutr..

[53] Ying T.S., Sarma D.S., Farber E. (1981). Role of acute hepatic necrosis in the induction of early steps in liver carcinogenesis by diethylnitrosamine. Cancer Res..

[54] Hong E.-J., Levasseur M.-P., Dufour C.R., Perry M.-C., Giguère V. (2013). Loss of estrogen-related receptor alpha promotes hepatocarcinogenesis development via metabolic and inflammatory disturbances. Proc. Natl. Acad. Sci. USA.

[55] Yu L.-X., Ling Y., Wang H.-Y. (2018). Role of nonresolving inflammation in hepatocellular carcinoma development and progression. NPJ Precis. Oncol..

[56] Mantovani A., Allavena P., Sica A., Balkwill F.R. (2008). Cancer-related inflammation. Nature.

[57] Scaffidi P., Misteli T., Bianchi M.E. (2002). Release of chromatin protein HMGB1 by necrotic cells triggers inflammation. Nature.

[58] Qin L.X. (2012). Inflammatory immune responses in tumor microenvironment and metastasis of hepatocellular carcinoma. Cancer Microenviron..

[59] Tian Z., Hou X., Liu W., Han Z., Wei L. (2019). Macrophages and hepatocellular carcinoma. Cell Biosci..

[60] Cassetta L., Pollard J.W. (2018). Targeting macrophages: Therapeutic approaches in cancer. Nat. Rev. Drug Discov..

[61] Ruffell B., Coussens L.M. (2015). Coussens, Macrophages and therapeutic resistance in cancer. Cancer Cell.

[62] Galdiero M.R., Bonavita E., Barajon I., Garlanda C., Mantovani A., Jaillon S. (2013). Tumor associated macrophages and neutrophils in cancer. Immunobiology.

[63] Noy R., Pollard J.W. (2014). Tumor-associated macrophages: From mechanisms to therapy. Immunity.

[64] Jiang J., Wang G.-Z., Wang Y., Huang H.-Z., Li W.-T., Qu X.-D. (2018). Hypoxia-induced HMGB1 expression of HCC promotes tumor invasiveness and metastasis via regulating macrophage-derived IL-6. Exp. Cell Res..

[65] Karin M., Greten F.R. (2005). NF-kappaB: Linking inflammation and immunity to cancer development and progression. Nat. Rev. Immunol..

[66] Lin Y., Higashisaka K., Shintani T., Maki A., Hanamuro S., Haga Y., Maeda S., Tsujino H., Nagano K., Fujio Y. (2020). Progesterone receptor membrane component 1 leads to erlotinib resistance, initiating crosstalk of Wnt/beta-catenin and NF-kappaB pathways, in lung adenocarcinoma cells. Sci. Rep..

[67] Cahill M.A., Jazayeri J.A., Kovacevic Z., Richardson D.R. (2016). PGRMC1 regulation by phosphorylation: Potential new insights in controlling biological activity. Oncotarget.

[68] Natarajan A., Wagner B., Sibilia M. (2007). The EGF receptor is required for efficient liver regeneration. Proc. Natl. Acad. Sci. USA.

[69] Xia H., Dai X., Yu H., Zhou S., Fan Z., Wei G., Tang Q., Gong Q., Bi F. (2018). EGFR-PI3K-PDK1 pathway regulates YAP signaling in hepatocellular carcinoma: The mechanism and its implications in targeted therapy. Cell Death Dis..

[70] Fuchs B.C., Hoshida Y., Fujii T., Wei L., Yamada S., Lauwers G.Y., McGinn C.M., Deperalta D.K., Chen X., Kuroda T. (2014). Epidermal growth factor receptor inhibition attenuates liver fibrosis and development of hepatocellular carcinoma. Hepatology.

[71] Lanaya H., Natarajan A., Komposch K., Li L., Amberg N., Chen L., Wculek S.K., Hammer M., Zenz R., Peck-Radosavljevic M. (2014). EGFR has a tumour-promoting role in liver macrophages during hepatocellular carcinoma formation. Nat. Cell Biol..

[72] Tsai H.-W., Ho C.-L., Cheng S.-W., Lin Y.-J., Chen C.-C., Cheng P.-N., Yen C.-J., Chang T.-T., Chiang P.-M., Chan S.-H. (2018). Progesterone receptor membrane component 1 as a potential prognostic biomarker for hepatocellular carcinoma. World J. Gastroenterol..

[73] Lee S.R., Kwon S.W., Kaya P., Lee Y.H., Lee J.G., Kim G., Lee G.-S., Baek I.-J., Hong E.-J. (2018). Loss of progesterone receptor membrane component 1 promotes hepatic steatosis via the induced de novo lipogenesis. Sci. Rep..

