# Supplementary material for: Loss of PGRMC1 Delays the Progression of Hepatocellular Carcinoma via Suppression of Pro-Inflammatory Immune Responses
Source: Cancers (Basel). 2021 May 18;13(10):2438. doi: 10.3390/cancers13102438 (PMC8157610; doi:10.3390/cancers13102438)
Supplement: Supplementary file 1 [file cancers-13-02438-s001.zip › cancers-1194539-supplementary.pdf]

Supplementary Figures

Loss of PGRMC1 delays the progression of hepatocellular carcinoma via suppression of pro-inflammatory immune responses

Sang R. Lee[1#], Jong Geol Lee[2#], Jun H. Heo[1], Seong Lae Jo[1], Jihoon Ryu[1], Globinna Kim[2], Jung-Min Yon[2], Myeong Sup Lee[3], Geun-Shik Lee[4], Beum-Soo An[5], Hyun-Jin Shin[1], Dong-Cheol Woo[2], In-Jeoung Baek[2*], and Eui-Ju Hong[1*]

#: These authors equally contributed to the study.

[*]Address correspondence to:
In-Jeoung Baek, DVM, PhD
Department of Convergence Medicine
Asan Medical Center, University of Ulsan College of Medicine
88, Olympic-ro 43-gil, Songpa-gu, Seoul 05505, Korea
Phone: +82-2-3010-2798; Fax: +82-2-3010-4197; Email: ijbaek@amc.seoul.kr

Eui-Ju Hong, DVM, PhD
College of Veterinary Medicine, Chungnam National University
99 Daehak-ro, Yuseong-gu, Daejeon 34134, Korea
Phone: +82-42-821-6781; Fax: +82-42-821-8903; Email: ejhong@cnu.ac.kr

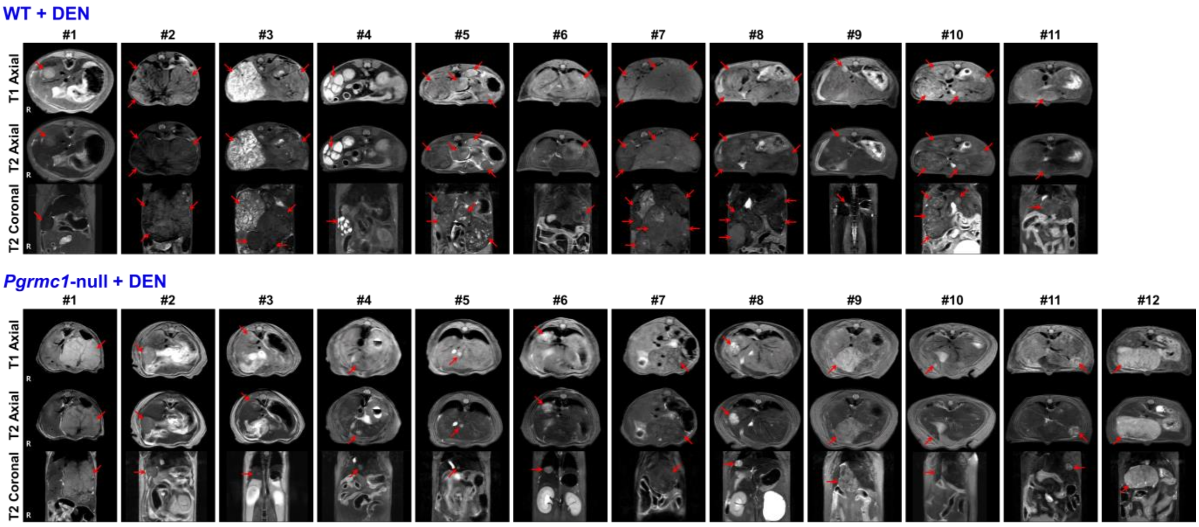

Figure S1. Magnetic resonance images (T1w [axial], T2w [axial and coronal]) of HCCs in HCC-bearing WT (n=11) and *Pgrmc1*-null (n=12) mice. Arrows denote the tumors.

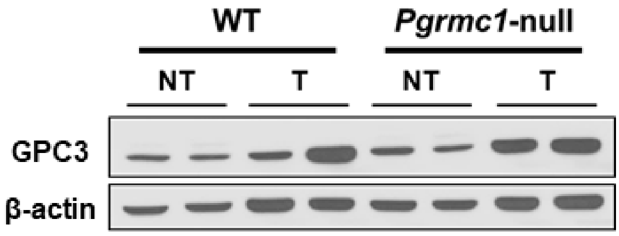

Figure S2. Western blot analysis of Glypican-3 (GPC3) in non-tumor lesions and tumors of WT and *Pgrmc1*-null mice. β-actin was used as an internal control. NT, non-tumor; T, tumor region

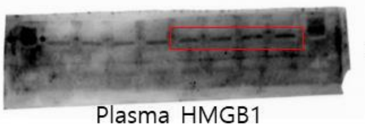
Fig. 3C
Plasma HMGB1

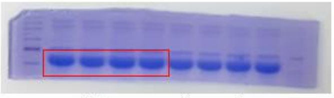
Fig. 3C
Plasma albumin

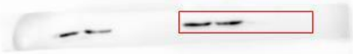
Fig. 4A
PGRMC1

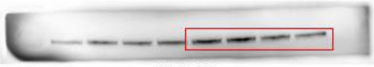
Fig. 4A
EGFR

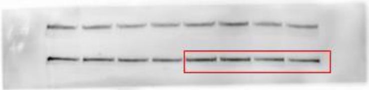
Fig. 4A
pEGFR

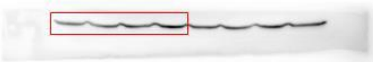
Fig. 4A
β-actin

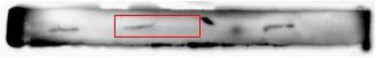
Fig. 4C
PGRMC1

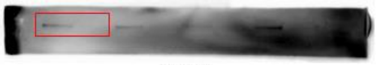
Fig. 4C
EGFR

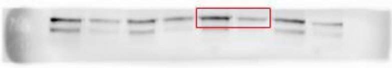
Fig. 4C
pEGFR

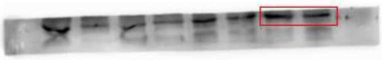
Fig. 4C
β-actin

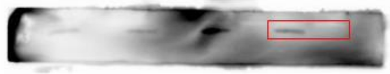
Fig. 4D
PGRMC1

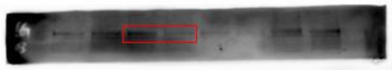
Fig. 4D
EGFR

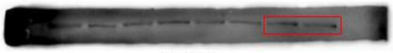
Fig. 4D
pEGFR

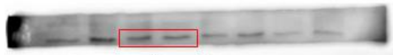
Fig. 4D
β-actin

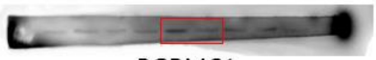
Fig. 6A
PGRMC1

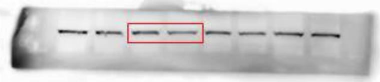
Fig. 6A
EGFR

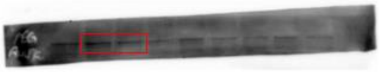
Fig. 6A
pEGFR

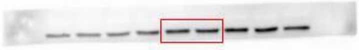
Fig. 6A
IκBα

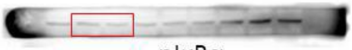
Fig. 6A
pIκBα

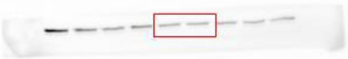
Fig. 6A
NF-κB (p65)

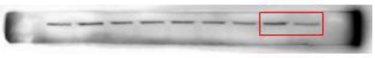
Fig. 6A
pNF-κB (p65)

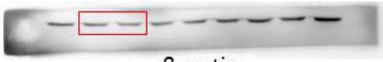
Fig. 6A
β-actin

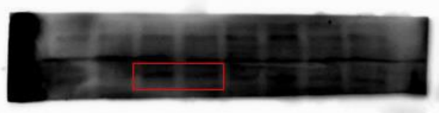
Fig. 6C
PGRMC1

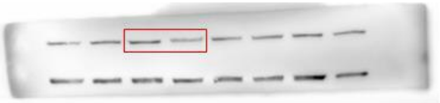
Fig. 6C
EGFR

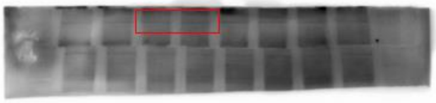
Fig. 6C
pEGFR

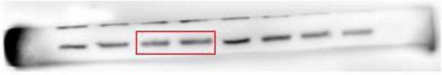
Fig. 6C
IκBα

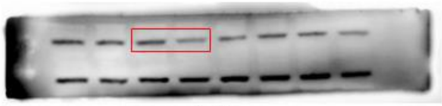
Fig. 6C
pIκBα

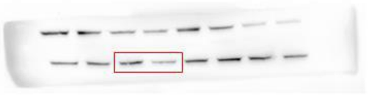

Fig. 6C

NF-κB (p65)

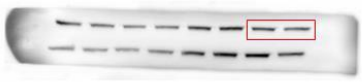

Fig. 6C

pNF-κB (p65)

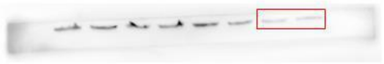

Fig. 6C

β-actin

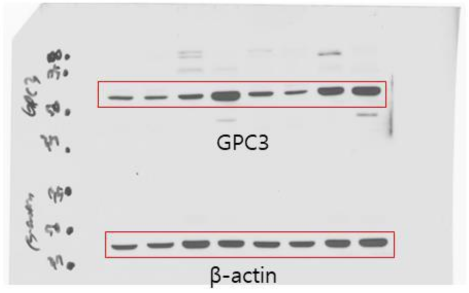

Fig. S2

Figure S3 Full blot images.

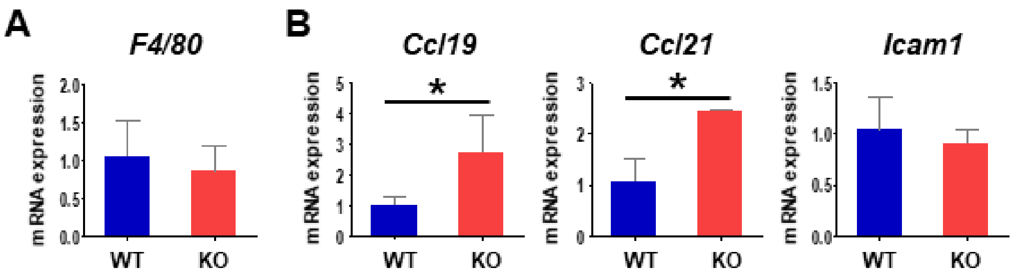

Figure S4. mRNA expression levels of *F4/80*, *Ccl19*, *Ccl21*, and *Icam1* in the livers of DEN-injected WT and *Pgrmc1*-null mice. The expression levels were normalized to that of *Rplp0* and expressed relative to the WT group. *p<0.05 in Student's t-test.

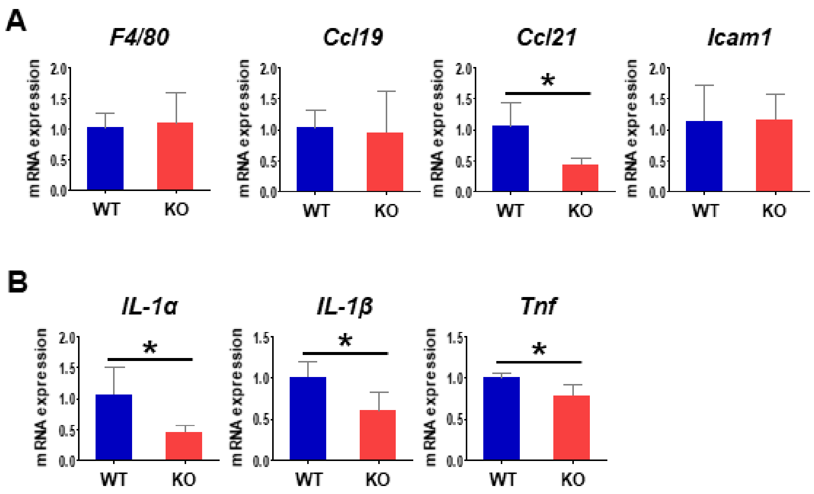

Figure S5. mRNA expression levels of *F4/80*, *Ccl19*, *Ccl21*, *Icam1*, *IL-1α*, *IL-1β*, *and Tnf* in the HCC of DEN-injected WT and *Pgrmc1*-null mice. The expression levels were normalized to that of *Rplp0* and expressed relative to the WT group. *p<0.05 in Student's t-test.

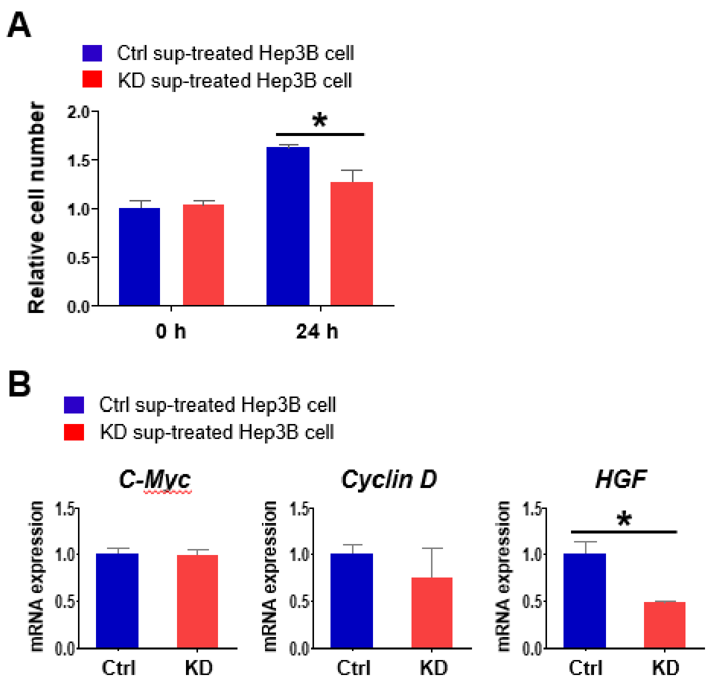

Figure S6. Cancer cell proliferation measured after treatment of Raw264.7 cell culture supernatant. (A) Cell number counted by trypan blue staining in 0 and 24 h treatment of Raw264.7 cell culture supernatant to Hep3B cells. FBS was excluded in Hep3B cell culture medium. Cell culture supernatant was collected from Control (Ctrl) or Pgrmc1-siRNA (KD)-transfected Raw 264.7 cells treated with necrotic debris for 3 h. (B) mRNA expression levels of *C-Myc*, *Cyclin D, and HGF* in Hep3B cells cultured with Raw 264.7 cell culture supernatant. Cell culture supernatant was collected from Control (Ctrl) or Pgrmc1-siRNA (KD)-transfected Raw 264.7 cells treated with necrotic debris for 3 h. Cell culture supernatant was incubated for 3 h in Hep3B cells. *p<0.05 in Student's t-test.

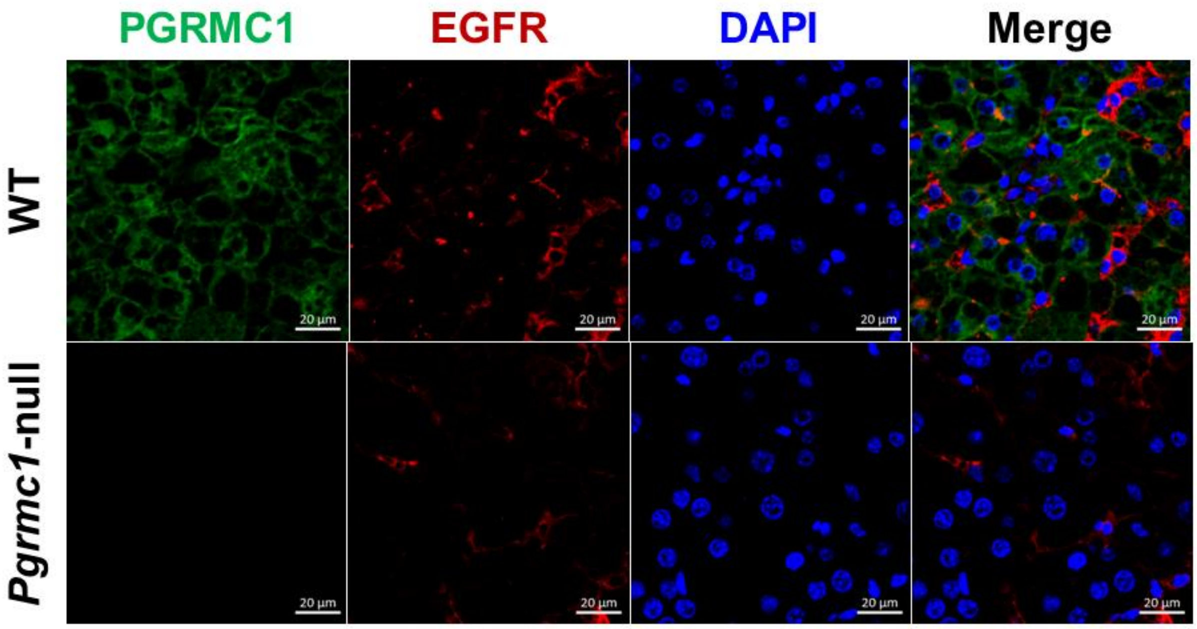

Figure S7. Co-immunostaining of PGRMC1 and EGFR in the HCC of WT mice and *Pgrmc1*-null mice. PGRMC1 (green) and EGFR (red) were stained with the corresponding antibodies. DAPI (blue) was used for nuclear staining.

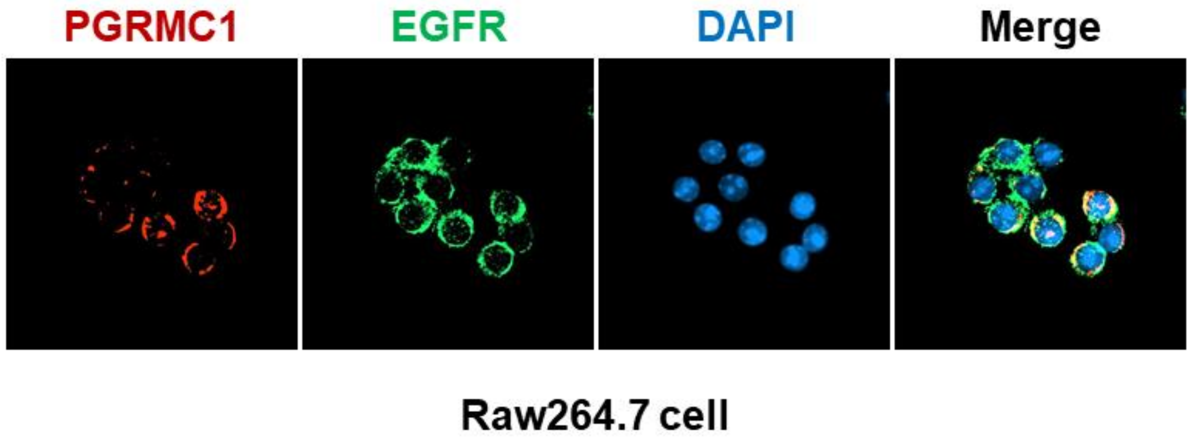

Figure S8. Co-immunostaining of PGRMC1 and EGFR in Raw 264.7 cell. PGRMC1 (red) and EGFR (green) were stained with the corresponding antibodies. DAPI (blue) was used for nuclear staining.
